# ‘The power of universality is that everybody is treated the same’: Exploring the possibilities and limitations of a universal approach to stigma reduction among BBV/STI sector stakeholders in Australia

**DOI:** 10.1186/s12954-025-01388-5

**Published:** 2026-01-19

**Authors:** Elena Cama, Emily Lenton, Adrian Farrugia, Gemma Nourse, Kate Seear, Amy Kirwan, Caitlin Douglass, Sophia Schroeder, Timothy R. Broady, Mark Stoové, Virginia Wiseman, Carla Treloar

**Affiliations:** 1 Centre for Social Research in Health, UNSW Sydney, Sydney, NSW 2052 Australia; 2 Australian Research Centre in Sex, Health & Society, La Trobe University, Melbourne, VIC Australia; 3 Deakin Law School, Deakin University, Melbourne, VIC Australia; 4 Burnet Institute, Melbourne, VIC Australia; 5 School of Public Health and Preventive Medicine, Monash University, Melbourne, Australia; 6 Kirby Institute, UNSW Sydney, Sydney, NSW Australia; 7 London School of Hygiene & Tropical Medicine, London, UK

**Keywords:** Stigma, Health care, Interventions, Blood-borne viruses, Sexually transmissible infections

## Abstract

**Background:**

There are increasing calls for cross-cutting approaches to reducing stigma in health systems that do not silo specific identities, conditions, or practices. In our previous work, we proposed a ‘universal precautions’ approach to addressing stigma and its negative effects (Treloar et al. in Harm Reduct J 19(1):74, 2022), whereby health systems assume that all people who enter a health service might be concerned about being treated negatively or excluded in some way. This paper explores the possibilities and limitations of such an approach to stigma reduction by canvassing key stakeholder perspectives.

**Methods:**

Qualitative interviews were conducted with 20 key stakeholders with extensive experience working within the alcohol and other drugs, blood-borne viruses, and sexually transmissible infections sectors. Participants were asked to reflect on the concept of a universal precautions approach to stigma reduction, including its acceptability, feasibility, utility in practice, and key challenges.

**Results:**

Although interview participants strongly advocated for a need to address all stigma within health care, there were mixed attitudes towards the use of the term, ‘universal precautions’. Some participants believed it would be useful to draw on a lexicon more familiar to health workers, while others expressed ambivalence and confusion about the term. Participants reflected on the possibilities of universality, in treating everyone with respect and providing non-judgemental care, while also emphasising that different client populations have specific needs that must be addressed. Many participants emphasised strongly that such an approach would need to be embedded at a systemic level, including having support from executive leadership, to address the structural forces that (re)produce stigma. Participants emphasised that centring the voices and perspectives of people with lived experience of stigma is integral to any approach to reducing stigma in health systems.

**Conclusions:**

Findings highlight the various possibilities of adopting a universal approach that recognises the diversity and intersections in experiences of stigma. This research informs the development and implementation of novel stigma reduction initiatives in health care settings.

## Background

Stigma is a concept which has received significant theoretical and empirical attention. There are several different approaches to conceptualising stigma. Goffman’s (p. 3) foundational work describes stigma as an ‘attribute that is deeply discrediting’ [2]. Link and Phelan described stigma as a process of steps, involving the co-occurring processes of distinguishing and labelling differences, associating these differences with negative attributes (i.e., stereotyping), separation according to difference (i.e., ‘us’ vs. ‘them’), status loss, and discrimination [3]. Importantly, within this approach, each step is shaped through social, economic, and political power differentials that allow one group to devalue and exclude another. This latter point is emphasised by the work of Parker and Aggleton, who highlight that stigma is a process which (re)produces power and control [4]. Reflecting a ‘macro turn’ in stigma scholarship, these approaches call for examination of the structural forces that scaffold processes of exclusion [5]. In the context of health, stigma can be understood as a social process through which people may be devalued and excluded based on perceived or actual health conditions, as well as practices or aspects of identity that receive broader societal stigma and are associated with that health condition [6]. 

There are multiple levels at which stigma occurs; individual, interpersonal, organisational or institutional, and structural. Individual stigma refers to the processes in which individuals respond to experiences of stigma, such as through attempts to conceal their stigmatised identity or through self-stigma [7]. Interpersonal stigma refers to interactions between the broader social group and stigmatised individuals and can include the attitudes and behavioural intentions that people, such as health workers, hold towards stigmatised groups of people [8]. Institutional and structural stigma have received significantly less attention in the stigma literature compared to individual and interpersonal stigma [9]. Sometimes discussed as a problem ‘hidden in plain sight’ (p. 50) [10], these types of stigma refer to how stigma manifests within socio-cultural norms, discourse, and institutional policies, and within taken-for-granted processes, all of which act to restrict the rights and opportunities for stigmatised people [11, 12]. In the context of health care, stigma can manifest as underfunding and under resourcing, denial of care or inequitable access to care, poor quality of health care, and coercive and paternalistic approaches to health care [8, 13]. Others have argued that stigma is a fundamental cause of health and health care inequalities, which cannot be explained (or remedied) by the availability of services [14, 15]. Although some scholars differentiate between institutional and structural stigma, we use these terms interchangeably in recognition of the ways in which state-based systemic discrimination can act as a barrier to the provision of and access to health care.

The impacts of stigma have been widely documented. Stigma can lead to reduced self-esteem, poorer quality of life, lower social support, greater psychological distress, and suicidal ideation and attempts [16–20]. Stigma is a known barrier to health care, with research finding that people who experience stigma are less likely to seek testing or care, as well as having lower uptake of treatments and of retention in care [16, 21–23]. It has also been found to be associated with increased symptoms, longer illness duration, and poorer physical health outcomes, the latter of which is potentially attributable to delays in accessing care due to stigma [16, 17, 20, 24]. This can increase the burden on health care resourcing through increased use of emergency and primary health care services [25]. 

There have been significant efforts in recent years to develop and trial programs and interventions to address stigma in health care settings. Scholars have suggested a number of key components or principles for successful stigma reduction programs. Nyblade, Mingkwan and Stockton argue for three core principles of HIV stigma reduction: (1) to address immediately actionable drivers of stigma (e.g., fear of infection and misinformation or lack of knowledge), (2) to centre affected groups in the response, and (3) to engage opinion leaders and create partnerships with affected groups [26]. Researchers have also categorised key stigma reduction intervention types: information-based, skills and capacity building, contact and partnership, counselling and support groups, biomedical, and structural interventions [26–28]. These efforts have tended to take a siloed approach to stigma (often mirroring health systems’ siloed approaches to managing, treating, and funding stigmatised health conditions themselves), focusing on addressing stigma in health care towards specific identities, conditions, or practices [29]. For instance, interventions targeting HIV stigma in health care have tended to use information and skills building, along with contact with people living with HIV, to improve the attitudes and behaviours of health workers towards people living with HIV [30]. Many of these interventions have shown short-term positive changes in attitudes towards specific identities, conditions, or practices [27, 28, 30]. However, there are many different conditions, identities, and practices that could benefit from stigma reduction interventions, and thus taking siloed approaches to stigma within health care may only respond to small pieces of the puzzle. Siloed approaches also require repeated interventions tailored to specific conditions, identities, and practices, and are therefore time and resource intensive.

As other researchers have noted, ‘While the etiology of stigma may differ between conditions and, sometimes, cultural settings, the manifestations and psychosocial consequences of stigma and discrimination are remarkably similar […] the core of stigma is social in nature’ (p.2) [31]. People may also be living with one or more stigmatised identities, health conditions, or practices [32] and may face overlapping or intersectional stigma or the ‘total synchronistic influence of various forms of oppression which combine and overlap’ (p. 24) [33]. Thus, cross-cutting approaches to stigma that move beyond siloed approaches to specific identities, conditions, and practices are needed to build feasible and sustainable stigma reduction interventions in health care settings [34]. In a recent paper, members of our research team proposed taking a ‘universal precautions’ approach to stigma and stigma reduction [1]. The term ‘universal precautions’ has historically referred to the range of practices for hygiene and infection control which have been designed to be applied consistently to all health care patients or clients to prevent exposure to blood and bodily fluids [35]. These practices have been further applied to stigma reduction in relation to HIV [36–40]; where health workers treat all patients the same regardless of their known or assumed HIV status, enabling a reduction in likelihood of potential infection, as well as shifting away from focusing on individuals as a source of risk towards focusing on practice as a source of risk. In our 2022 paper, we draw from the work of Knaak, Patten and Ungar (p. 864) in viewing stigma as a quality-of-care problem and ‘strategically colluding with and leveraging already well accepted processes and priorities for health-care providers and organisations’ [41]. Thus, we proposed leveraging ‘a strategic collusion between the equity pillar of health care quality, stigma reduction and […] universal precautions.’ (p. 3) [1]. Our approach seeks to develop interventions that support health systems to recognise the diversity of people who access health services, with each person potentially concerned about experiencing stigma in relation to any number of identities, conditions, or practices they may have experienced or engaged in over their lifetime.

Following the publication of our commentary, the Australian Government Department of Health funded a four-year project to further develop this idea and implement a trial of a universal approach to stigma within health care settings across the country. Given this is a new approach to stigma reduction, our first step was to scope the perspectives of key stakeholders and experts, beginning within our home ground of the BBV and STI sector, to explore what we might seek to gain, or lose, by taking a universal approach to stigma reduction. We chose this sector as a focus because BBVs/STIs are highly stigmatised and noting the salience towards BBVs/STIs in relation to the root causes of stigma (concealability, course, disruptiveness, aesthetics, origin, and peril) [42]. That is, blood-borne viruses such as HIV may be stigmatised both due to perceptions of peril around the virus itself (e.g., fear of infection, misinformation) and negative attitudes (such as disgust) towards potential transmission routes (e.g., injecting drug use, unprotected sex) [43]. 

In this paper, we explore how key stakeholders responded to the proposed universal precautions approach to stigma reduction, including acceptability of the concept and terminology, how this approach could be applied in practice, and challenges for its application. Our aim is to explore the possibilities and limitations of a universal approach to stigma.

## Methods

### Sample and procedure

Our team conducted semi-structured qualitative interviews with 20 key informants who had extensive experience working in the BBV/STI sector and who had knowledge and expertise to interrogate a complex new conceptual approach to stigma reduction. Participants were purposively invited to ensure a balance of insights and experiences across health settings, states and territories, location (urban and regional), experience working in the BBV/STI sector, and from community-led organisations and roles. The interviews ranged from 60 to 90 mins in duration and were conducted over Zoom. All participants provided written consent to participate prior to the interview.

Key informants were assigned an interview number, setting description and role, and were not assigned pseudonyms. Demographic characteristics of the sample are presented in Table 1. Participants were employed in a range of positions, including policy, senior management, program design and management, service provision, and advocacy. Lived and/or living experience of the participants were also included if they were in identified roles, services, or if they disclosed this during the interview. Participants were not reimbursed for their time. Ethics approval was obtained by the UNSW Human Research Ethics Committee and La Trobe University Human Research Ethics Committee (reference: HC220669).

The semi-structured interview guide was developed by the research team and informed by the aims of the research. Participants were prompted to discuss and reflect on the terminology of universal precautions in the context of stigma reduction, identify tensions, and consider what may be overlooked or missed by removing the focus on identities, practices and health conditions. The interviews asked participants to describe what constitutes quality health care, to speak to existing policies, frameworks, and strategies that have the capacity to structurally embed stigma reforms, and identify barriers and enablers to stigma reform. These interviews informed the following stages of the project, including interviews with clinical and non-clinical health workers and co-design workshops. The purpose of this stage of the project was to focus on acceptability of this approach and to generate ideas or models to guide the co-design phase of the research.

**Table 1:** Characteristics of the stakeholder sample (*N* = 25)

	*N*
*Gender*	
Female	12
Male	7
Other	1
*State*	
Australian Capital Territory	1
New South Wales	7
Northern Territory	2
Queensland	1
South Australia	2
Victoria	6
Western Australia	1
*Organisational reach*	
State	15
National	5
International	2
*Setting*	
Tertiary, hospital (Tertiary)	5
Community health	5
Primary health	1
Government	4
Professional association	2
Higher education institute	2
Research institute	1
*Focus area*	
Viral hepatitis	4
HIV/sexual health	1
All fields	15
*Sector experience*	
1–7 years	1
8–15 years	2
16–22 years	3
23–30 years	7
31 + years	7

### Data analysis

Interviews were audio-recorded and professionally transcribed. Transcripts were checked for accuracy and deidentified by the interviewers (EL, GN). A coding framework was developed in alignment with the research project’s aims, in consultation with the research team, research partners, and the project advisory group. Interviews were coded in QSR NVivo 14 by the interviewers using an inductive approach [44]. Themes and issues identified during this process were incorporated into the coding framework. To ensure consistency, code usage was cross-checked by EL and GN. The framework was structured to code stigma at individual, institutional, and systemic levels. It also coded for existing policies, procedures, regulatory and legal frameworks, and practices that stigma interventions could be embedded. Additionally, it considered intersectional and multiple stigmas, including those related to gender and sexuality, culture and diversity, and health conditions.

This paper focuses on key stakeholder perspectives around the acceptability, feasibility, and utility of adapting a well-established concept—that of universal precautions—for addressing stigma. For the purposes of this paper, acceptability is understood to be the perception among stakeholders that the intervention is agreeable, while feasibility refers to the extent to which stakeholders believe the intervention could be successfully carried out in healthcare settings [45]. We use the term utility to explore the extent to which stakeholders perceived taking a ‘universal’ approach to stigma to be beneficial or useful.

## Results

### Acceptability and debates around terminology

Although all participants strongly advocated for a need to address stigma within health care, there were mixed responses towards the appropriateness of the terminology ‘universal precautions’ in the context of stigma. Participants in support of the term suggested that linking a familiar concept for health workers to stigma reduction was a logical step or, as one participant called it, a ‘no brainer’ (P4, tertiary, programs and policy). Universal precautions in their original conception have previously shown benefits in addressing HIV stigma [36–40]. As we have explained elsewhere, ‘if you treat every situation the same in terms of the potential for infection, then the particularities of an individual are decentred as a source of risk or contagion concern, and hence the conditions and assumptions that give rise to stigma might also be shifted.’ (p. 3) [1]. Thus, it might be logical to extend the concept to stigma more broadly.

The history of why universal precautions [came] into being and a lot of that was to do with the HIV response. So, it does get back to good preparedness of the health workforce and then even if somebody did disclose [their HIV status], you know, again [health workers were prepared] in how to respond to that, to assure the person of why things are done a certain way, in a health care setting. (P6, government, policy)

I love it, but I love it because […] that whole notion of universal precautions is a no brainer. It’s like okay, ‘If we treat everyone the same at the highest standard to protect all of us’ then you are going to capture everyone. (P4, tertiary, programs and policy)

For these participants, they perceived that one of the benefits of using this terminology was that it would be easier for health workers to accept the concept, given they were already familiar with the terminology. Other scholars have similarly suggested that stigma within health care may be best achieved by using existing logics and lexicons, with which health workers are familiar [8, 41]

Everyone knows what universal precautions are […] so, when you’re trying to […] the concept to someone, but relating to a different issue, such as in this instance, stigma and discrimination, it’s going to really help to sort of encourage people to pick this concept up a lot faster. (P5, government, policy)

However, others expressed some confusion about how this phrasing could apply to stigma and stigma reduction. For instance, one participant was unclear on how the application of the concept would go beyond the treatment of blood and management of infection risk.

I am still struggling with how you would apply it […] I understand the concept, and I thought really, that’s really what should be happening everywhere is that people should be treating all [patients] the bloody same […] and having the same practices, that should just be standard, but I think in terms of stigma in the health workforce, in terms of viral hepatitis, it’s more than just the treatment of blood […] I’m not sure how universal precautions relates to that, you know, I don’t know, I might be missing something. (P2, community, advocacy)

Here, although P2 sees the potential in applying clinical terminology to a social construct, they indicate that there might be some nuances that need to be addressed in transposing it to its new context of stigma-reduction.

Further, some participants expressed concerns about the use of the term ‘precautions’ given that the term could be perceived as alarming or frightening; or, indeed, could itself be stigmatising. P4, while supportive of the extending this terminology to stigma, expressed concern that doing so may have unintended consequences. They suggest that, for people not familiar with the existing use of the term, that ‘the word “precaution” is kind of a little bit alarming, it’s like what are we frightened of? Why do we need to be cautious?’ (P4, tertiary, programs and policy). These participants suggested that using this term might inadvertently position patients or clients as a ‘threat’ or ‘problem’, or someone to take ‘precautions’ *against*. As P8 said, ‘That’s the concern I have with that kind of approach, because it says, “Someone here is a problem and we just have to be careful with them”. (P8, tertiary, infectious diseases physician).

Other participants were more ambivalent about the term, with one saying, ‘I’m not sure it sets me on fire’ (P14, community, program/service delivery, lived and/or living experience). These participants were unsure of how this concept would work in practice and were especially concerned that it might be ‘politically’ challenging to get the health system to apply this framing of stigma reduction, particularly as it would be difficult to develop a shared understanding of stigma and what a universal approach to stigma means.

I think politically it would be interesting to see how that would run, to be honest, like to [basically say] we have got a universal concept of stigma and there are lots of people that are impacted by stigma. I think it would be difficult […] because I think politically it would be really hard […] I don’t know, I am just probably speaking out of turn, but [my concern is] whether you would get a position where everyone agreed. (P18, professional association, policy)

As P1 argues below, one of the challenges of using this terminology in practice stems from the way universal precautions, as currently conceived, are not applied universally. According to them, the reason for inconsistent application was that some health conditions and identities are especially stigmatised, underpinned by stereotypes and categories of risk. Therefore, they had witnessed health workers, for example, using gloves for a person who has tattoos due to the perception of risk related to unsafe tattooing, while not wearing gloves for an older person without tattoos. This observation is consistent with previous stigma research in the BBV and STI field, which has found that excessive or inappropriate hygiene and infection control procedures are one of the ways in which stigma may manifest within health care settings [46–49]. P1 went on to reflect on the move away from the term ‘universal precautions’ to one of ‘standard precautions’, indicating that perhaps a similar evolution should occur when thinking about stigma.

The terminology, ‘universal precautions’ with regard to infection control is no longer used and they changed it to ‘standard precautions’ […] I liked the framing of this in terms of stigma, because it should be universal and maybe in time, we’ll end up talking about it being standard precautions for stigma prevention, because it should just be baseline, best practice, it shouldn’t be anything different, shouldn’t be anything new, shouldn’t be anything that we have to teach people, but maybe we do have to teach people about the impact of their behaviour on people’s health seeking and the way they deal with their health issues. (P1, research institute, program design)

Overall, participants saw the utility of a broad cross-cutting approach to stigma reduction in health care, although there were debates about whether ‘universal precautions’ was the most appropriate terminology to frame such an approach. P1 above highlights one of the central tensions of this framing—that we seek to universally recognise concerns about stigma for those accessing health care, while also respecting difference. Difference may exist in relation to people’s experiences of stigma and/or their specific health care needs or concerns, and how these are addressed in the health care setting may therefore require a nuanced approach.

## A universal precautions approach in practice

Participants reflected on what a universal approach to stigma should look like and how it could be implemented. One of the core principles of such an approach proposed by many participants was the notion of treating everyone the same and with basic respect.

The power of universality is that everybody is treated the same. (P6, government, policy)

Treating everybody of every age, gender, ethnicity in the same way, with basic respect. (P1 research institute, program design)

The underlying basis for taking a universal approach to stigma recognises a need to ensure people do not feel judged when accessing health care. A universal approach would highlight that health workers and systems should treat everyone well regardless of who they are, their practices, or health conditions. Participants recognised that people make judgements and that asking health professionals not to hold these judgements might not be practical or feasible.

I mean the thing that first comes to mind [with universal precautions] is non-judgemental [care], right, […] accepting that we all have judgements about others, we make them all the time. […] But as a health care professional, you [need to] kind of suspend that and not allow it to affect the quality of care that [you’re] providing. (P3, community, advocacy, lived and/or living experience)

Leave your prejudices at the door and work with the person in front of you in a totally non-judgmental way and a respectful way. (P5, government policy)

Recognising these challenges, it was argued that a universal precautions approach to stigma should instead require health professionals to actively manage how their preconceptions might impact the quality of care they provide. Participants therefore argued that reflective practice should be central to implementing a universal approach to stigma, and this was seen as a feasible request given that reflective practice is something already expected of and undertaken by health workers. Although there is no consistent or accepted definition, reflective practice can be understood as a cognitive skill that involves health professionals’ examining their own beliefs, values, and practices in order to learn from their own experiences and adapt their future practices to ensure improved patient outcomes [50]. Considering these definitions, the participants in our research expected health professionals to reflect critically on their own beliefs, values, or judgements about patients and adjust their practices to ensure that these do not impact on the quality of care provided. As the following excerpts highlight, participants also believed that empathy for other people is integral for reflexive practices aimed at reducing stigma.

I think it’s about hearing people; you know if they are having negative health care experiences or if they are challenging in their behaviour, I think that is about being able to stop and hear people, hear them out and take what they are saying on board. (P12, tertiary, manager)

We have to teach health care workers, we have to teach them that and it’s not necessarily just teaching, it’s helping them to understand the impact of their behaviour, reflecting on their behaviour and developing an empathy and understanding of people without necessarily having to have that next step, which is judgment or assessment of the worthiness. (P1 research institute, program design)

Participants reflected on how health worker attitudes and beliefs, including cultural and religious beliefs, can influence their care practices. Given the varied personal attitudes and beliefs of health workers, some participants were unsure of how a universal approach written into policy could be effectively or feasibly implemented. Nevertheless, participants emphasised the importance of policy in setting the expectation for health workers to ‘reach for the absolute ultimate high standard right across the board, we treat everyone the same, we do it well, we do it with respect, we do it properly’. (P4, tertiary, programs and policy).

So what would a universal approach be? […] I mean, it should be that people, no matter what the choices are in life, [as a health worker] you treat them well and you know, you’re there to provide a health care service, but […] as we all know, people’s attitudes come into it, and people are judgmental and so I’m not sure how a policy or how a universal precautions policy works in that setting. (P2, community, consultant, lived and/or living experience)

Here, P2 indicates that a ‘universal approach’ to stigma reduction might not influence health worker’s attitudes, which they suggest might be difficult to change.

When making sense of universal precautions, participants drew on existing concepts within health care, such as person-centred care, to highlight how health workers should sensitively address health concerns that the individual presents with.

It’s person-centred, and completely non-discriminatory, regardless of any element, any criteria, that’s what anybody should expect walking into a health care facility […] it’s around recognising the health problem, the presenting problem within the context of someone’s life and so both their condition and [any other contextual factors] should be you know, be sensitively addressed, so very, very about the person, very focused on the person. (P6 government, policy)

When reflecting on the application of a universal precautions approach to stigma in practice, participants cited non-judgemental practices as core. As the following participant demonstrates, this could be as simple as a health professional actively asking the individual why they are there, to open the opportunity for discussion of health concerns.

To […] have wholesome holistic care, so you know even letting the patient tell you why they are there today you know. First thing my GP always says to me, ‘What are you doing here today, what can I help you with?’ so you feel like there is an opening to have that discussion. I just think some of the solutions will be really simple […] Do you know what I mean? It’s not hard. (P15, government, policy)

Participants also argued that the ways health workers ask for information during appointments was another issue to consider in a universal approach to stigma. They argued health workers should only ask consumers for the information they directly need for the purposes of any testing and care they provide and to be transparent about the forms of care they recommend. From this position, health workers must remain sensitive to the experiences and impacts of stigma, and ensure that these inform care interactions, including by not discussing stigmatised practices unless they are essential for care [51].

I think it actually just comes back to not tying identity to practices, right? You know, it’s not assuming because someone looks one way when you see them that, you know, they’re seeing clients of a particular gender or doing particular things with them, you know. Asking what you actually need to know to get tests done, and then if people want to share more with you, that’s fine, but otherwise, just like really sticking to the facts about, you know, practices and what’s needed on the basis of that. […] It comes back to transparency as well. Just being transparent about whatever you’re doing and why you’re doing it. […] People like to know what’s happening to them. (P20, professional association, policy)

I think at a high level principle, we’re talking about your interaction with the patient in front of you, you’re providing them with the access to the best possible health care that you can give them and [ensuring] that any information that they provide you within that interaction or that you can glean about that person, that you apply no judgment to that and to whatever attributes or experiences […] may have culminated to form the person that’s in front of you, you are just treating the person in front of you on [the basis of] whatever health conditions they come to you for support with. (P5, government, policy)

Centring the expertise of people with lived experience stigma and building partnerships with affected communities are central to stigma reduction initiatives [26, 52]. Importantly, centring lived experience in stigma reduction requires including people with lived experience in the initial development and delivery of interventions. Previous researchers have found significant positive effects of what is referred to as ‘contact’ interventions, which facilitate interactions between health care workers or other communities and people with lived and living experience, with the aim of building empathy and dispelling myths and stereotypes [53, 54]. In our study, participants similarly pointed to the importance of centring lived experience in a stigma reduction intervention through, for example, training of health workers. This could include having people with lived experience speak to health workers as part of education and training, as well as ensuring people with lived experience are consulted with as part of intervention development.

I do think the power of having people with lived experience, sharing their knowledge and enlightening staff about what it actually feels like, I think has the potential to shift that change. (P13, tertiary, research and education)

A lot of empowerment also comes from […] consultation groups with staff, listening to staff, listening to attitudes, perhaps allowing opportunities for the client group and the staff to come together. (P7, tertiary, infectious diseases physician)

Ensure that you get patients involved at the very beginning, not just at the end, to adjudicate on the outcomes or your toolkit, for example, […] If you’re not involving the patients, you’re going to miss out on some key aspects (P3 community, advocacy, lived and/or living experience).

Given the range of conditions, practices, and identities which are stigmatised [32], it may not be feasible to include representation of all of these voices. This highlights a key tension within a universal approach; deciding which voices are heard and facilitating appropriate representation and diversity in the voices of people with lived experience.

Reflecting established responses to stigma that develop recommendations for specific conditions, identities, or practices [26], rather than universally applicable practices, some participants expressed concerns about whether a universal approach to stigma reduction would or could address the needs of particular populations. While P1, for instance, was generally favourable of a universal approach to stigma, they reflected that ‘certain populations require a nuanced approach so, it’s not a one-size-fits-all’. Despite these queries about feasibility, they nevertheless endorsed ambitious efforts to address stigma universally.

I can see the merits, but I think in practice it’s going to be really difficult for a lot of those reasons that I’ve just said, because you’re talking about, you know, really complex situations where you’re going to have to factor in a lot of things, but absolutely that doesn’t mean you shouldn’t [try]. (P19, government, policy)

Broadly, participants perceived the core underlying principle of a universal approach is to treat everyone with basic respect. While participants suggested the notion of treating everyone the ‘same’, this largely referred to services setting standards for appropriate and welcoming care, while still recognising the need for tailored practices specific to conditions, people, or concerns. Participants perceived that drawing on existing concepts and practices, such as person-centred care and reflective practice, would be useful and feasible in implementing a universal approach.

### Addressing structural forces that (re)produce stigma

One of the key concerns raised by our participants was the need to ensure a universal approach to stigma addressed structural forces that (re)produce stigma and health inequities. Echoing P19’s questions about the relationship between addressing the specificities of different forms of stigmas and a ‘one-size-fits-all’ approach, P9 questioned whether a universal approach would struggle to address inequity:

What I would argue quite strongly is that an equal application is not equal, particularly when it comes to stigma and discrimination […]. I am sure you have seen it, there is a little cartoon that I often use when I am teaching medical students that says, ‘equity is not equality’ and for me, from my perspective universal precautions […] brings that concept of equality to the conversation, I think, and I maybe oversimplifying it, but it doesn’t bring *equity*. […] it doesn’t address the inequities. (P9, Education, program manager, emphasis added)

To address these concerns, participants emphasised that any stigma reduction efforts need to be embedded in a health service at a systemic level such as through policies, reflective practice, and staff supervision practices. In this way, addressing health care systems, rather than individual practices, was positioned as likely to generate more support and traction.

So, if it was taken as a systems approach, then I think you would get more traction, but there’s a lot of layers to getting that support getting that buy-in, [facilitating an] agreement on the type of development that you would need in place, but also how we would report and that’s, again, a challenging area, because there’s so many, there’s so much reporting done, that large service providers would really push back on it. So, it’s a simple idea, but a lot of complexity in how it would be implemented. (P6, government, policy)

I think it is, it’s getting it into people’s scope of practice, but this needs to be supported by senior leadership, registrations systems, supervision, and strong support […] in policy, and embedded in other systems that support cultural change, and have the structural support of time. (P14, community, program/service delivery, lived and/or living experience)

Participants suggested that achieving this could involve specific messaging that places responsibility on the health system to ensure that a zero-tolerance approach to stigma is upheld in policy and practice.

How do we make people feel that they are not going to be judged? We are here as a health service for all of your body, all of you, every part, you know, every part that makes you a being […] And that’s where I kind of go back to that you know, “I am non-judgemental” or “I am a champion for truth in health” or something, I don’t know, just something that gets the message out to the punter but also puts the pressure on the system. (P16, tertiary, health promotion)

While the support of executive and leadership was positioned as vital for a universal precautions approach to be feasible, in keeping with the ‘total facility approach’ [55], our participants emphasised that all health service staff need to be included in stigma reforms. Having support at the executive and leadership level was seen as a powerful tool for influencing acceptability among staff. Participants also believed that staff needed to be able to see that they also have a role to play in addressing stigma.

Someone once asked a cleaner about their job and they said, ‘I make the hospital safe and clean so people can get good health care’ and it’s about your place in making health care better right […] It’s how people see themselves as part of that picture, I think is very important. (P15, government, policy)

Previous stigma reduction interventions have tried to harness influential individuals within health services to act as ‘champions’ of stigma reduction [36, 38, 55]. Some participants similarly pointed to the need to identify influential people who could garner support for a universal approach to stigma.

If we are saying ‘Well, yes we’ll have a universal approach […] [so we have to ask] what does that look like? How is it executed in different communities? And then who were the champions of it?’ (P14, community, program/service delivery, lived and/or living experience).

Although a universal approach to stigma reduction largely focuses on health worker practices and the systems underpinning health services, one participant did reflect on the importance of also informing consumers about their rights. However, beyond informing consumers about their rights within these settings, it is also crucial to create an environment in which complaints processes are accessible and transparent, and for consumers to feel safe using them [56, 57].

I think it’s also about making people understand their rights […] actually clearly understanding that they don’t have to disclose or where they should disclose, what’s right, what’s acceptable behaviour. If they haven’t received acceptable behaviour, what do they do to actually call it out? So, I think that’s probably an area that hasn’t been well covered. Certainly, that latter bit, about if you’re not happy with the behaviour then what do you do to call it out? (P13, tertiary, research and education)

Finally, participants drew attention to the broader political context that shapes health care service provision. Overall, they argued that stigma reduction is a political project beyond specific programs and initiatives. Broadening the notion of ‘universal’ to include political action, advocating for the inclusion of stigma reduction as a goal in national and state strategic health care policies, key performance indicators in the mandates of services, as well as the inclusion of stigma reduction in quality and safety standards (stigma is not currently included in the Australian Quality and Safety Standards).

What does quality health care look like and how do we define quality health care in the context of minimising or eliminating episodes of stigma and discrimination in a health care setting? We need to come to some kind of agreement of what a definition of that is, then insert that into our national and state strategic documents that are committed to by governments and then we ask that governments actually put that into policy directives with mandated compliance across all services within their domain and that would then cascade down through the system and give us a lever to do some of these other things that are going to be your enablers like workforce development, like community education about pathways to report episodes of stigma and discrimination, and all these other things. So you have to get that consistency at that high level in the first instance. (P5, government, policy)

As participants have highlighted, a universal precautions approach to stigma alone may be limited in its ability to address the structural forces that (re)produce stigma. It is evident that broader systemic change is needed, and this project presents an opportunity to advocate for a focus on stigma at the national and state policy level.

## Conclusion

The aim of this article was to explore the possibilities and limitations of translating the notion of ‘universal precautions’ into ambitious efforts to address stigma in health care ‘universally’. Through interviews with key stakeholders in health care, we examined the acceptability and utility of the concept overall alongside perceptions of the most appropriate terminology, feasibility and applicability in practice, and the potential challenges for implementation. Some limitations of the research should be noted. This study sample consisted of stakeholders based in the Australian context. Stakeholders were also working within the BBV/STI sector, and this group was selected because due to the salience towards BBVs/STIs in relation to the root causes of stigma [42]. Future research would benefit from an examination of perspectives towards a universal approach to stigma reduction in other health care contexts and in the context of other stigmatised identities, conditions, and practices.

While our analysis suggests that key stakeholders had mixed responses to the terminology of ‘universal precautions’, others argued that mobilising existing health care logics and lexicons was a strength [8, 41]. Overall, this debate was focussed on most effectively developing an ambitious stigma response and, in this sense, these stakeholders generally supported the utility of and were invested in the development of a broad universal or cross-cutting approach to reducing health care stigma in Australia. What such an approach might look like in practice was less clearly articulated by stakeholders in this study. Participants largely proposed principles that they believed were important within a universal approach to stigma; namely, the notion of treating everyone the same and with basic respect. Participants also highlighted the importance of ensuring that people who access health care do not feel judged. Although these principles could be written into health care policies and guidelines and may already be reflected to some degree in policies relating to equity and access or anti-racism, how to ensure that they are enacted remains a considerable challenge of this approach [58]. 

What also remains unclear is the extent to which a universal approach to stigma could address one of the core principles of stigma proposed by Nyblade, Mingkwan and Stockton [26]; that is immediately actionable drivers of stigma, such as fear of infection and misinformation or lack of knowledge. Taking a universal approach to stigma might be limited in the extent to which an intervention can feasibly improve knowledge of or address negative attitudes towards specific identities, conditions, or practices. However, even interventions which have taken on such a task of addressing knowledge and attitudes towards specific health conditions like HIV or mental illness have demonstrated limited long-term and sustained change among health care staff [9, 30, 59, 60], Key stakeholders interviewed in this study suggested that within a universal approach to stigma, we might call for health workers to set their beliefs or judgements about others aside for the purpose of delivering quality care and to exercise reflective practice by recognising how belief systems can negatively impact on patients and subsequent health outcomes. Thus, the application of a universal approach in practice involved treating people the same, with respect, non-judgemental care, and with ongoing reflective practice. Existing concepts, such as reflective practice and person-centred care, were seen as useful for providing health care workers with a framework to practice a universal approach to stigma [61]. As such, a universal approach cannot be reduced to treating everyone the ‘same’. Rather, this approach holds certain standards for appropriate and welcoming care as universal while acknowledging that tailored practices may be required for specific conditions, people, or concerns. In this sense, acknowledging and working with difference is itself a ‘universally’ applied principle of this approach.

In keeping with the principle of centring lived experience in stigma reduction [26, 52, 62], our interview participants strongly advocated for building partnerships with people with lived experience of stigma related to a range of identities, conditions, and practices. The power of including lived experience voices in education for health workers to build empathy and understanding was one regular suggestion. Indeed, previous research has found that interventions which facilitate contact between health workers and people from affected communities is effective in building empathy and addressing negative attitudes and misconceptions that health workers might hold towards affected communities [53], and to communities that were not the target of the original contact [63, 64]. However, a key tension to grapple with in a ‘universal’ approach is how to ensure appropriate representation and diversity in the voices of people with lived experience included in any intervention.

One of the key challenges to a universal approach to stigma raised by participants was whether such an approach would be able to address the structural forces that (re)produce stigma. Previous research has drawn attention to the ways in which stigma may become embedded within the socio-cultural norms and institutional policies of the health care system [8,11,12], often enabled by laws and legal processes such as those that criminalise and stigmatise certain behaviours, like drug use [65]. Reflecting this, systematic reform is an integral part of a universal approach to addressing stigma. In this sense, interventions mobilised within a universal agenda would include all staff from all levels of the organisation, from those working in executive and leadership positions to those delivering health care, working in reception, and so on. With such an ambitious agenda, the need for adequate funding must be considered. In this sense, the ‘universal’ includes not just addressing all health care clients and encounters as potentially shaped by stigma but advocating for systemic and sustained change. This might involve, for example, advocating for the inclusion of stigma in state and national policies and guidelines, and through national quality and safety standards, as well as incorporating lived experience in high-level systems design. Such a remit is, of course, a necessarily ambitious and long-term project, however, the alternatives are not acceptable: that is, to be immobilised in the face of complexity and challenge at hand, or to choose some sectors in which to take action over others when all are deserving of appropriate, welcoming, and inclusive health care.

## Data Availability

Individual-level data contain sensitive information and are not suitable for a public repository due to ethical restrictions. Data is available in de-identified form upon reasonable request and pending ethics approval.
